# Secondary osteosarcoma arising after treatment for childhood hematologic malignancies

**DOI:** 10.3109/03009730903177340

**Published:** 2009-12-08

**Authors:** Atsushi Okada, Masahito Hatori, Masami Hosaka, Munenori Watanuki, Eiji Itoi

**Affiliations:** ^1^Department of Orthopaedic Surgery, Tohoku University School of MedicineJapan; ^2^Department of Orthopaedic Surgery, South Miyagi Medical CenterJapan

**Keywords:** Acute lymphoblastic leukemia, non-Hodgkin's lymphoma, osteosarcoma, secondary cancer

## Abstract

Secondary osteosarcoma arising after the treatment of hematologic malignancies other than Hodgkin's lymphoma is rare. We report two cases of secondary osteosarcoma arising after treatment for childhood hematologic malignancies (non-Hodgkin's lymphoma and lymphoblastic leukemia). A 10-year-old boy, at the age of 3, was diagnosed with non-Hodgkin's lymphoma. He received chemotherapy, radiation, and bone-marrow transplantation and then was in complete remission. At 6 years, he complained of increasing pain of the right thigh and was diagnosed with osteoblastic osteosarcoma. A 26-year-old man, at the age of 6, was diagnosed as having acute lymphoblastic leukemia (ALL). He received chemotherapy, radiation, and peripheral blood stem cell transplantation (PBSCT). At 11 years after PBSCT, he visited with the complaint of left lumbar swelling. He was diagnosed with chondroblastic osteosarcoma. In both cases alkaline phosphatase (ALP) had already increased prior to the onset of the symptom. We should rule out secondary osteosarcoma at the abnormal elevation of ALP during clinical follow-up of patients after treatment of childhood hematologic malignancies.

## Introduction

Osteosarcoma is one of the most common secondary malignant neoplasias following childhood malignancies (1). Ewing's sarcoma, retinoblastoma, rhabdomyosarcoma, and Hodgkin's lymphoma are the most frequent primary malignancies causing secondary osteosarcoma (2,3). However, secondary osteosarcoma arising after the treatment of hematologic malignancies other than Hodgkin's lymphoma is rare (4). We report two cases of secondary osteosarcoma following treatment of childhood hematologic malignancies.

While previous reports mostly focused on the primary malignancies during childhood followed by secondary osteosarcoma, or on the possible causing agent related to this entity, there have been no reports concerning early detection of secondary osteosarcoma. Alkaline phosphatase (ALP) is considered to be involved in calcification of the bone matrix and protein synthesis associated with bone matrix production (5). There is fairly positive correlation between the activity of plasma total ALP and the osteoblastic activity in primary osteosarcoma patients (6–8). The clinical value of this marker in secondary osteosarcomas, however, has not been reported. In this study, we examined the time course of serum ALP expression in two patients with secondary osteosarcoma following childhood hematologic malignancies.

## Case report

### Case 1

**Figure 1.**
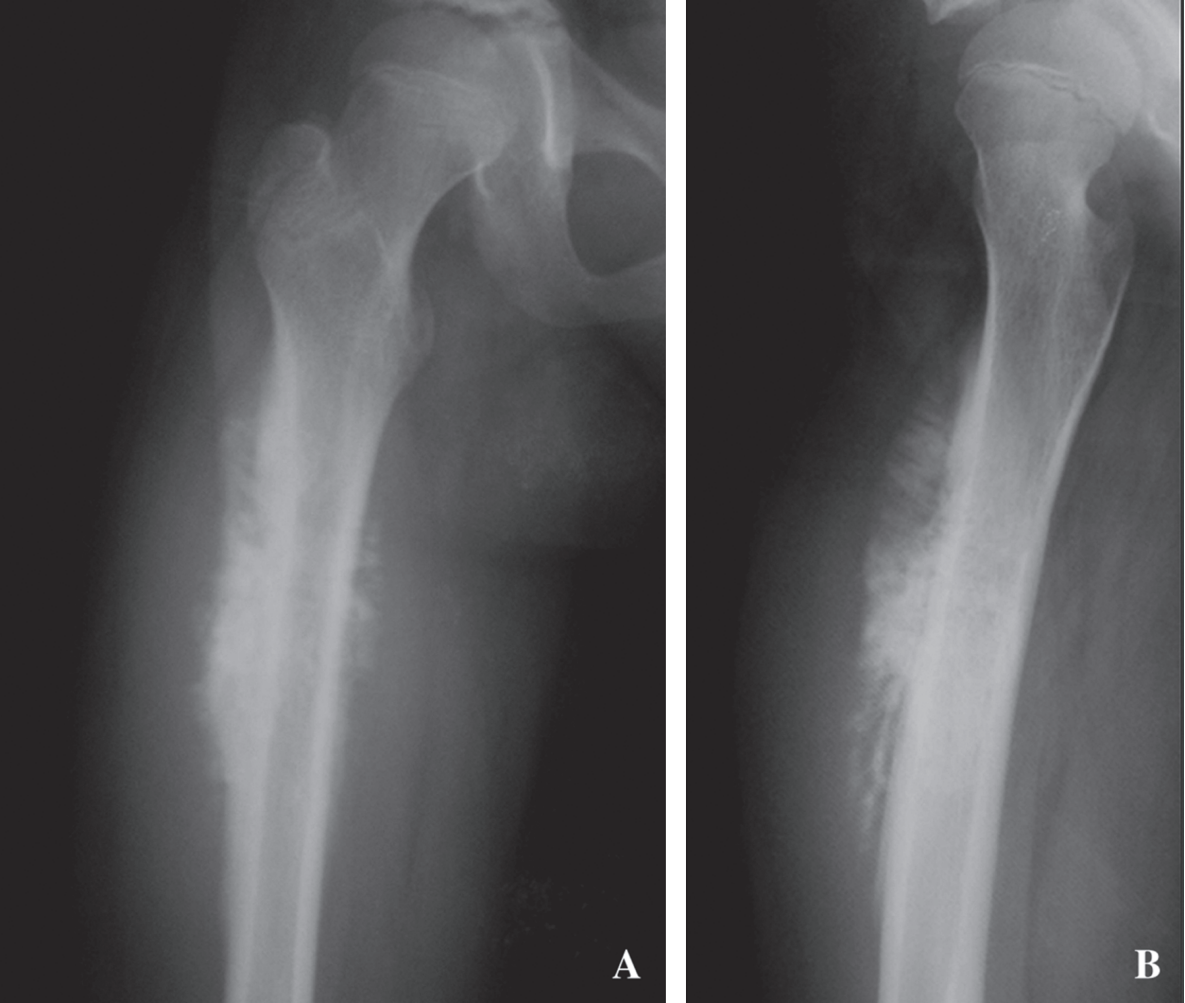
Case1. Plain radiograms of the proximal femur revealed a mixed sclerotic and lytic intramedullary lesion with the periosteal perpendicular spiculation with extensive periosteal reaction and “Codman's triangle” reaction.

**Figure 2.**
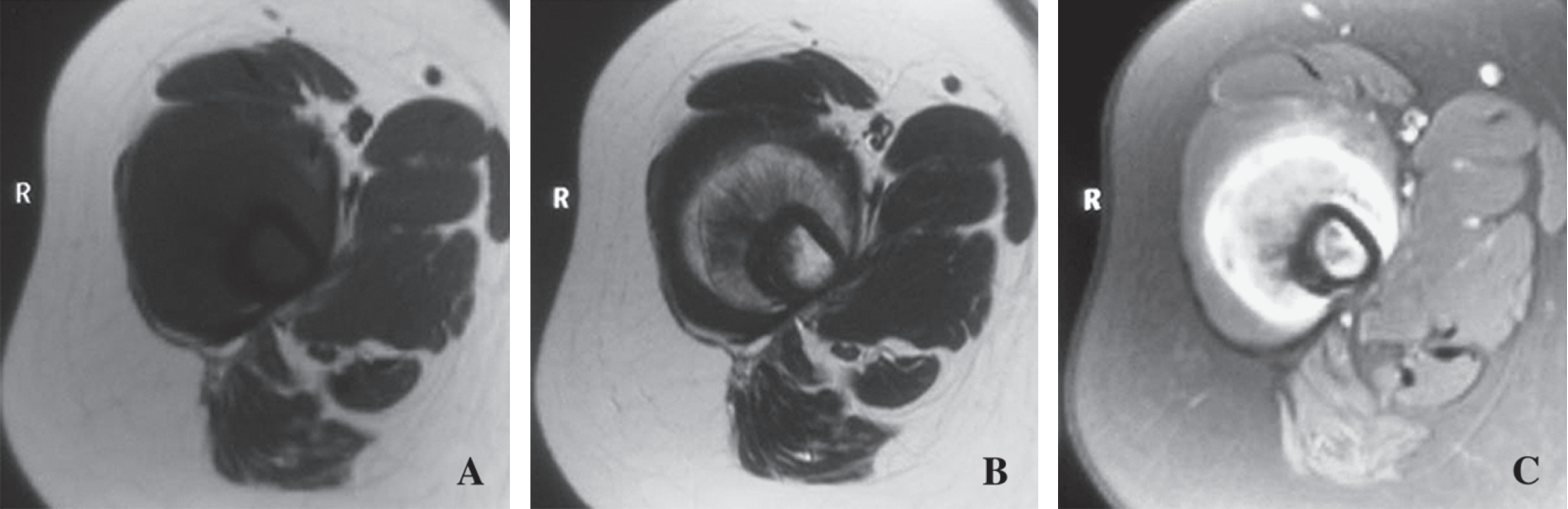
Case1. (A) MRI revealed an isointense lesion within the quadriceps femoris muscle on T1-weighed images. (B) T2-weighed images showed a heterogenous hyperintense lesion. (C) The lesion was enhanced heterogeneously on fat-suppressed T1-weighed images after gadolinium injection.

**Figure 3.**
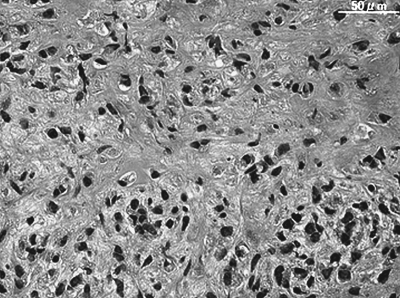
Case1. Histological appearances of the biopsied specimen demonstrated spindle or polygonal cells with atypical nuclei, eosinophilic amorphous osteoid and matured bone tissue.

A 10-year old boy, at the age of 3, was diagnosed with non-Hodgkin's lymphoma (gamma/delta T cell lymphoma). Chemotherapy was initiated according to Tohoku Childhood Leukemia Protocol. This consisted of induction with vincristine, methotrexate, and daunorubicin, followed by three courses of high-dose methotrexate and high-dose cytarabine. After the confirmation of remission by bone-marrow aspiration, he underwent allogeneic bone-marrow transplantation from his (Human Leukocyte Antigen)-identical brother. Since then he was in complete remission. At 6 years after the transplantation, the patient complained of increasing pain of the right thigh. Laboratory data showed that his ALP was 2802 IU/L (normal: 200–620). At 2 months prior to the onset of the symptom, serum ALP level had already elevated to an abnormally high level of 1604 IU/L. Plain radiograms of the proximal femur revealed a mixed sclerotic and lytic intramedullary lesion with extensive periosteal reaction (Figure 1). Magnetic resonance imaging (MRI) revealed an isointense lesion within the quadriceps femoris muscle on T1-weighted images. The lesion was enhanced heterogeneously on fat-suppressed T1-weighted images after gadolinium injection. T2-weighted images also showed a heterogeneous hyperintense lesion (Figure 2). Histological appearance of the biopsied specimen demonstrated spindle or polygonal cells with atypical nuclei, eosinophilic amorphous osteoid, and matured bone tissue (Figure 3). Histological diagnosis was osteoblastic osteosarcoma. Chemotherapy with Rosen T-20 was administered to the patient. After preoperative chemotherapy, wide excision of the tumor was performed. Postoperative chemotherapy was performed uneventfully, but the patient developed local recurrence and lung metastasis 15 months after the operation. Despite the irradiation to the thigh and chemotherapy, the recurrent tumor continued to grow. The patient died of respiratory failure 24 months after the operation.

### Case 2

**Figure 4.**
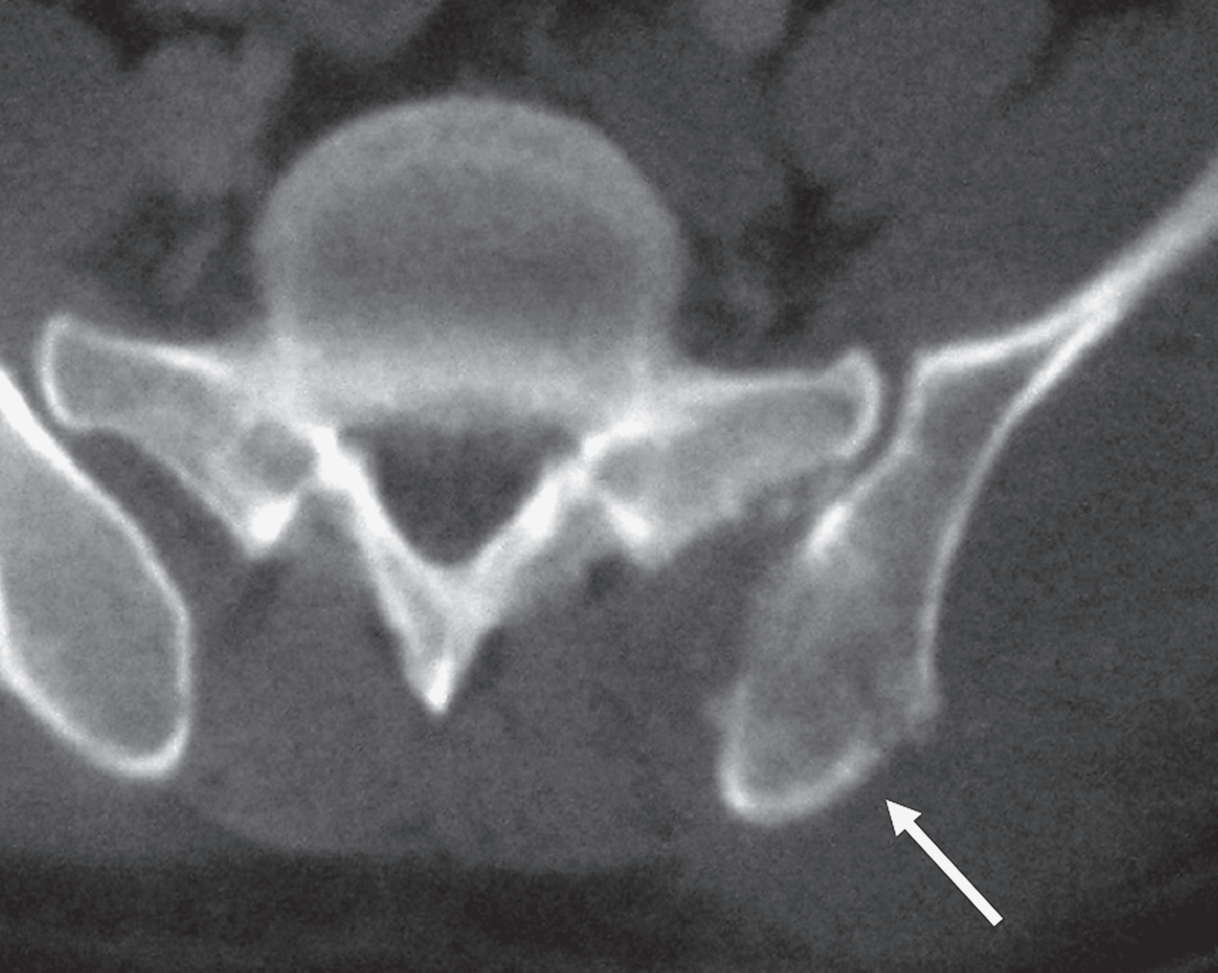
Case 2. Plain CT showed a lytic lesion in the posterior superior iliac spine (arrow).

**Figure 5.**
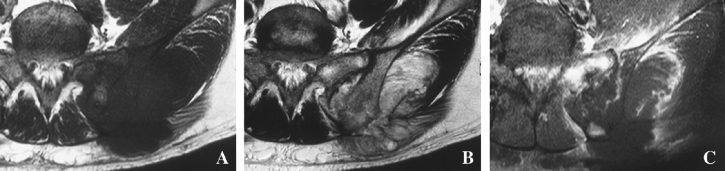
Case 2. (A) MRI showed a low-intense lesion around the left sacroiliac joint on T1-weighted images. (B) T2-weighed images confirmed a heterogenous hyperintense lesion. (C) The lesion was enhanced on fat-suppressed T1-weighed images.

**Figure 6.**
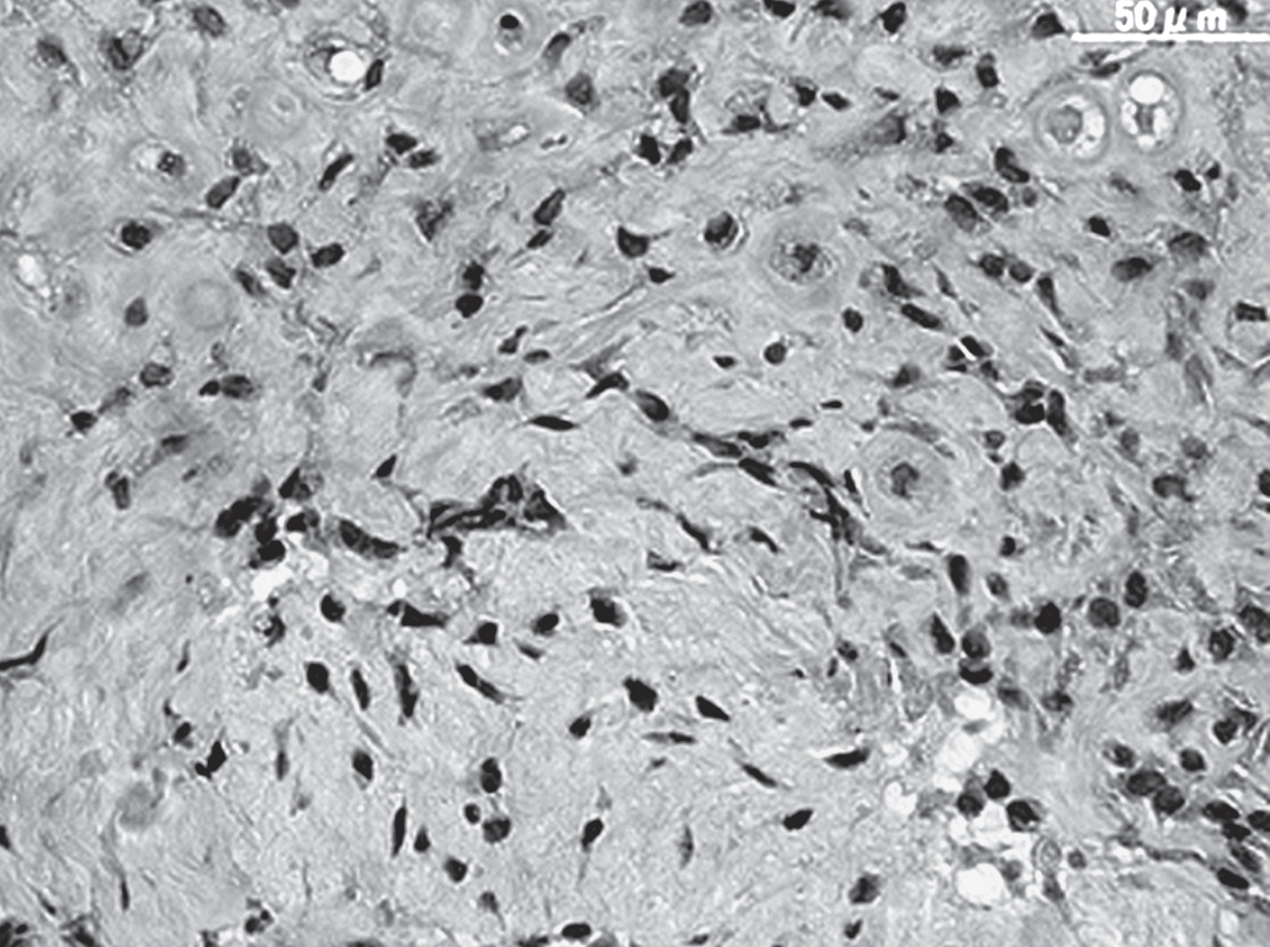
Case 2. Needle biopsy of the left iliac spine showed polygonal cells with hyperchromatic nuclei. The stroma consisted of chondroid and osteoid tissues.

A 26 year-old-male, at the age of 6, was diagnosed as having acute lymphoblastic leukemia (ALL). Chemotherapy was initiated according to Tohoku Childhood Leukemia 83B protocol consisting of 6-mercaptopurine, methotrexate, adriamycin, vincristine, and cyclophosphamide. After 5 years of chemotherapy, complete remission was achieved. Ten months later, at the age of 12, he noticed that his right testis was swollen. Biopsied specimen showed leukemic infiltration. After chemotherapy (prednisolone, vincristine, adriamycin, and asparaginase) for 3 years, irradiation to bilateral testis (total 26 Gy), total body irradiation (total 12 Gy), and peripheral blood stem cell transplantation (PBSCT), complete remission was obtained. At 11 years after PBSCT, he visited our department with the complaint of left lumbar swelling. Laboratory data showed ALP was 674 IU/L (normal: 112–330). At 3 months prior to the onset of the symptom, serum ALP level was already elevated to an abnormally high level of 559 IU/L. On plain computed tomography (CT), a lytic lesion in the posterior superior iliac spine was apparent (Figure 4). MRI showed a low-intensity lesion around the left sacroiliac joint on T1-weighted images. The lesion was enhanced on fat-suppressed T1-weighted images. T2-weighted images confirmed a heterogeneous hyperintense lesion (Figure 5). Needle biopsy of the left iliac spine showed polygonal cells with hyperchromatic nuclei. The stroma consisted of chondroid and osteoid tissues. Pathological diagnosis was chondroblastic osteosarcoma (Figure 6). At 2 weeks after the biopsy, the patient felt left leg pain. Abdominal CT showed loss of pooling of contrast medium in the left *inferior vena cava*, which suggested a thrombus. The patient died of pulmonary embolism 4 weeks after the biopsy.

## Discussion

The occurrence of a secondary malignant neoplasm in a child after or during treatment of a primary malignancy is the most ominous effect of the treatment (4). Osteosarcoma is one of the most common secondary malignant neoplasms following cancer treatment (4). An increased risk for developing osteosarcoma is associated with prior tumors such as retinoblastomas (9), Ewing's sarcomas (9,10), rhabdomyosarcomas (2,11), and Hodgkin's lymphomas (2,3,12). Osteosarcomas following hematologic malignancies other than Hodgkin's lymphoma, on the other hand, are rare (13–16). Newton et al. reported 52 cases of secondary osteosarcoma, which included 5 cases of Hodgkin's lymphoma as a primary cancer. Other malignancies were retinoblastomas (16 cases), Ewing's sarcoma (13 cases), rhabdomyosarcomas (5 cases), Wilms tumors (4 cases), and others (7 cases) (3). Le Vu et al. reported 32 cases of secondary osteosarcoma including 2 cases of Hodgkin's lymphoma and 2 cases of non-Hodgkin's lymphoma as primary malignancies. Others were Ewing's sarcoma (8 cases), rhabdomyosarcoma (6 cases), retinoblastoma (5 cases), neuroblastoma (2 cases), tumors of central nerve systems (2 cases), and others (5 cases) (2). Nygaard et al. reported that no osteosarcomas were detected in a population-based series of 8 second malignancies in 895 childhood ALL (13). None were detected among 43 second neoplasms in 9720 children with ALL in a study by Children's Cancer Study Group, no osteosarcomas among 16 second malignancies in 497 patients with non-Hodgkin's lymphoma, and only 1 out of 52 second neoplasms among 5006 ALL patients (14–16).

As for the treatment of secondary osteosarcoma, numerous studies have demonstrated the increased aggressiveness of, and the increased rate of mortality associated with, post-irradiation osteosarcoma compared with its naturally occurring counterpart (17). Sim et al. reported that the average duration of survival was 1.1 years after the diagnosis of post-radiation bone sarcoma in 55 patients at the Mayo Clinic. The poorer outcome for these patients may be due to a delay in the diagnosis, because many symptoms are thought to be related to primary diseases or radiation osteitis (18). Bechler et al. reported that of nine cases of secondary osteosarcoma only one patient had survived free of disease for 5 years. Most of the secondary tumors developed in the axial skeleton, were not amenable to complete operative excision, and were associated with pulmonary metastasis. They found that radiation and various chemotherapeutic agents had only palliative effects and did not affect the course of the disease appreciably (11). Our two cases were characterized by a poor outcome. In Case 1, despite chemotherapy and irradiation, the patient developed local recurrence and lung metastasis and died of respiratory failure. In Case 2, the patient died of thrombosis suddenly, probably due to tumor thrombus.

Several agents have been proposed as a cause for many secondary cancers (19). The role of radiation therapy in carcinogenesis of secondary malignant neoplasm is well known (3,12,20–22). Le Vu et al. reported that the risk of osteosarcoma was found to be a linear function of the local dose of radiation (2). Radiation-associated osteosarcoma has historically been associated with a poor prognosis, because they are typically locally invasive and high-grade (23).

Several studies have found an association between prior alkylating agent and anthracycline therapy and osteosarcoma (2,3). However, a causal relationship between chemotherapy alone and the development of a second malignant neoplasm in children has not been established (11). Bone-marrow transplantation, which commonly combines treatment with high-dose alkylating agents and total body irradiation, may be associated with second malignant neoplasms. Asai et al. reported a case of osteosarcoma 3 years after allogeneic bone-marrow transplantation (24). Bielack et al. also reported four cases of osteosarcoma following hematopoietic stem cell transplantation (25).

Both of our two cases were treated with chemotherapy including either anthracycline (daunorubicin) or alkylating agent (cyclophosphamide) and total body irradiation ahead of stem cell transplantation.

Although prior reports mostly focused on the primary malignancies during childhood associated with secondary osteosarcoma, or on the possible causing agents related to secondary osteosarcoma, there have been no reports regarding the early detection of the secondary osteosarcoma. Previous studies have shown that plasma total ALP activity raised to an abnormal level at the time of recurrence or metastasis in patients with osteosarcoma (6,26,27). Liu et al. reported that plasma bone-specific ALP levels of osteosarcoma patients at the time of recurrence were significantly higher than those of the patients without recurrence (28). Bramer et al. compared pre-chemotherapy ALP levels with those of post-chemotherapy levels in 89 cases of non-metastatic high-grade sarcoma and concluded that a pre-chemotherapy ALP above twice the normal level correlated with worse survival and that post-chemotherapy ALP correlated with survival and response to chemotherapy (29). Here in this report, we found that the elevation of the plasma level of ALP preceded the aggravation of the symptom. We should rule out secondary osteosarcomas at the elevation of ALP during clinical follow-up after treatment of malignancies.
